# Preparation, Structure, and Properties of Polystyrene-Microsphere-Reinforced PEG-Based Hydrogels

**DOI:** 10.3390/polym13162605

**Published:** 2021-08-05

**Authors:** Chen Zhang, Zhanping Zhang, Yuhong Qi

**Affiliations:** Department of Materials Science and Engineering, Dalian Maritime University, Dalian 116026, China; pudding@dlmu.edu.cn (C.Z.); yuhong_qi@dlmu.edu.cn (Y.Q.)

**Keywords:** polystyrene microspheres, polyethylene glycol, composite hydrogel, structure, property

## Abstract

To improve the mechanical strength and practicability of hydrogels, polystyrene microspheres with core–shell structure were prepared by the soap-free emulsion polymerization, polyethylene glycol hydrogels with polystyrene microspheres by the in-situ polymerization. The structure, morphology, roughness, swelling property, surface energy, and mechanical properties of the microspheres and hydrogels were investigated by Fourier transform infrared spectroscopy, scanning electron microscopy, transmission electron microscopy, confocal laser microscopy, swelling test, contact angle measurement, and compression test. The results showed that they have certain swelling capacity and excellent mechanical properties, and can change from hydrophobic to hydrophilic surface. The reason is that the hydrophilic chain segment can migrate, enrich, and form a hydration layer on the surface after soaking for a certain time. Introducing proper content of polystyrene microspheres into the hydrogel, the compressive strength and swelling degree improved obviously. Increasing the content of polystyrene microspheres, the surface energy of the hydrogels decreased gradually.

## 1. Introduction

Hydrogels are a gel system formed by hydrophilic polymers and water molecules with a three-dimensional interconnection network structure that can swell in water but not dissolve [1,2,3]. In 1960, Wichterle and Lim [4] found a soft, swelling, elastic and transparent material in the presence of a small number of crosslinking agent, and a certain number of water or an appropriate solvent during the polymerization of hydroxyethyl methacrylate monomer. This research result opened a prelude to the synthesis and application of hydrogels. Subsequently, the diverse of hydrogels emerged and developed rapidly. The hydrogels are widely used in tissue engineering [5,6,7], biomedical devices [8,9], microfluidic, optical actuators [10,11], and the marine industry [12,13] due to its high moisture, physiological fluid, and high elasticity. From the source of preparing hydrogels, they can be divided into natural macromolecule hydrogels and synthetic hydrogels; according to the different ways of crosslinking, they can be divided into physically cross-linked hydrogels and chemical cross-linked hydrogels; according to different sizes of hydrogels, they can be divided into macroscopic hydrogels, micron hydrogels, nano hydrogels, etc. [14].

It is a common phenomenon that the swelling property of hydrogels is accompanied by complex water evaporation and water absorption in the actual environment, which will lead to the weakening of hydrogels and loss of their original properties, such as mechanical properties. On the one hand, by grafting hydrogels onto carriers with high mechanical strength, the mechanical properties of hydrogels can be greatly improved. On the other hand, functionalized hydrogels can be obtained by grafting certain functional chains onto hydrogels. Ewaramm [15] copolymerized N-vinyl caprolactam onto guar gum, then it mixed with sodium alginate and cross-linked with glutaraldehyde to obtain a composite hydrogel, and applied the hydrogel to control release, which can effectively control the release of zidovudine (a drug for the treatment of AIDS) through pH and temperature. Zhuo [16] copolymer P(HEMA-co-MMA) obtained by copolymerization of Hydroxyethyl methacrylate (HEMA) with methyl methacrylate (MMA) can further improve the mechanical strength of hydrogels while maintaining the hydrophilic properties. Li [17] prepared a novel PAM-GO nanocomposite hydrogel using graphite oxide (GO) as a multifunctional crosslinking agent and acrylamide (AM) as a monomer. Compared with the traditional PAM hydrogel, the obtained hydrogel has excellent mechanical properties, elongation at break is more than 3000%, tensile strength up to 385 kPa, and excellent swelling property. Zhu [18,19] used photoactive polystyrene (PS) nanoparticles as a crosslinking agent to prepare a covalent crosslinking PAM-PS nanocomposite hydrogel with excellent mechanical properties and stability. The mechanical properties and swelling degree of the gel changed with the number of PS microspheres and monomers in the initial reaction solution. In addition to the interactions between the polymer and PS microspheres, hydrogen bonding between the polymer chains also plays an important role in gel formation.

Because there may be grafting, interpenetration, or ionic bonding between the core and shell of core–shell polymer microspheres, the wear resistance, water resistance, weather resistance, pollution resistance, radiation resistance, and tensile strength of the polymer can be significantly improved, and the film-forming temperature can be reduced and the processing property can be improved as well [20]. Similarly, core–shell microgels are special gels with a special structure formed by coating the gel shell layer on the gel core. Such gel particles have core and shell properties due to their composition and structure characteristics [21,22]. During preparation, according to the properties of the monomers and the feeding sequence, the microgels with normal core–shell structure or special-shaped core–shell structure can be formed [23,24]. Pelton [25] reported for the first time the preparation and characterization of a thermo-sensitive poly (N-isopropylacrylamide), namely PNIPAM microgel. They first used soap-free emulsion polymerization to prepare polystyrene (PS) as the core and PNIPAM as the shell hydrogel particles. Later, many people reported the preparation of PNIPAM nanohydrogels and copolymerized hydrogels containing PNIPAM blocks by different and similar methods [26,27]. Polyethylene glycol (PEG) is a kind of polymer with -CH_2_CH_2_O- as the structural unit. In its main chain structure, the oxygen atom has a high polarity, so this polymer has a high hydrophilicity. At present, the main chemical steps of preparing PEG-based hydrogels are to modify the hydroxyl terminal of PEG and introduce some controllable chemical crosslinking functional groups, gelation is then realized [28]. For example, ethylene is modified on the two ends of PEG by the esterification method, and the gel can be formed by olefin addition of mercapto group reaction in the presence of polymercaptan [29].

Due to the characteristics of simple preparation and excellent performance of nanocomposite hydrogels, polymer hydrogels are irreplaceable in many fields. However, at the present stage, the properties of polymer hydrogels also have some defects, such as poor mechanical properties and single function, which hinder their application to a certain extent. Therefore, the research on nanocomposite hydrogels will continue and open up a broader field of application.

To improve the mechanical properties of the hydrogel, novel nanocomposite hydrogels were prepared in this paper, which was designed that PEG hydrogel as a base and nano microspheres were used to reinforce PEG hydrogel. PEG-based nanospheres composite hydrogels were prepared by two-step method. Styrene (St), hydroxyethyl methacrylate (HEMA), and polyethylene glycol methyl ether methacrylate (PEGMA) were used as monomers to prepare nanometer microspheres with core–shell structures by soap-free emulsion polymerization of styrene. The chemical structure, morphology, swelling property, contact angle, surface energy, and mechanical compression property of PS-PEG hydrogels were tested and characterized to prepare nano-composite hydrogels with high compressive strength.

## 2. Materials and Methods

### 2.1. Materials

The reagent used in this study was styrene (St, CP), purchased from Tianjin Damao Chemical Reagent Co., Ltd. (Tianjin, China). Hydroxyethyl methacrylate (HEMA, AR), polyethylene glycol methyl ether methacrylate (PEGMA, AR), azobisisobutamidine hydrochloride (AIBA, AR), isphorone diisocyanate (IPDI, AR), 1, 4-butanediol (BDO, AR) and polyethylene glycol (PEG) with an average molecular weight of 2000 were purchased from Aladin Biochemical Technology Co., Ltd. (Shanghai, China). Polyether triol (HSH330, CP) was purchased from Jiangsu Hai‘an Petrochemical Plant (Hai’an, China). Xylene (AR) was purchased from Tianjin Tianli Chemical Reagent Co., Ltd. (Tianjin, China). Ethyl acetate (AR), potassium bromide (KBr, AR), and cyclohexanone (AR) were purchased from Tianjin Kemiou Chemical Reagent Co., Ltd. (Tianjin, China).

### 2.2. Synthesis Principle and Process Method

#### 2.2.1. Synthesis Principle

1. Chemical Reaction Principle of Preparing Polystyrene Microspheres

**Figure 1:**
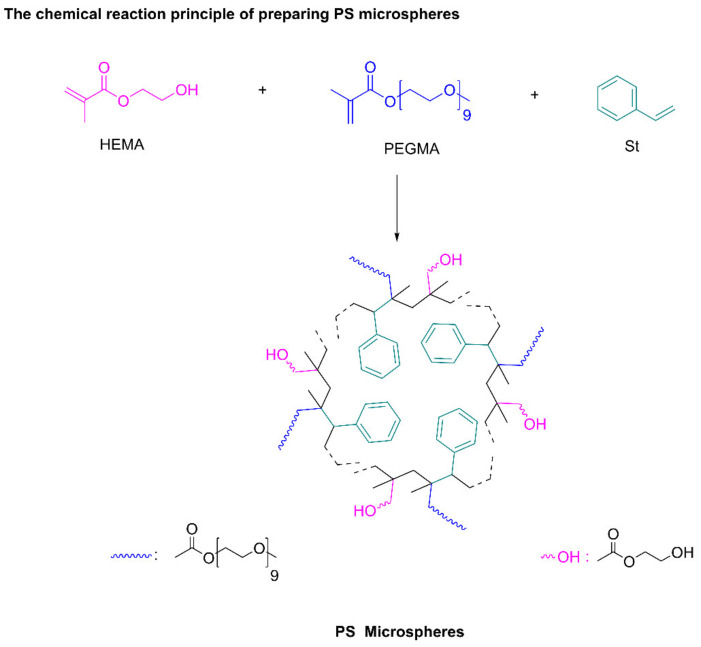
Chemical reaction principle of preparing PS microspheres.

Polystyrene microspheres were prepared by soap-free emulsion polymerization using styrene, HEMA and PEGMA as reaction monomers and AIBA as initiator, as showed in Figure 1.

2. Chemical Reaction Principle of Preparation of PS-NCO Intermediates

**Figure 2:**
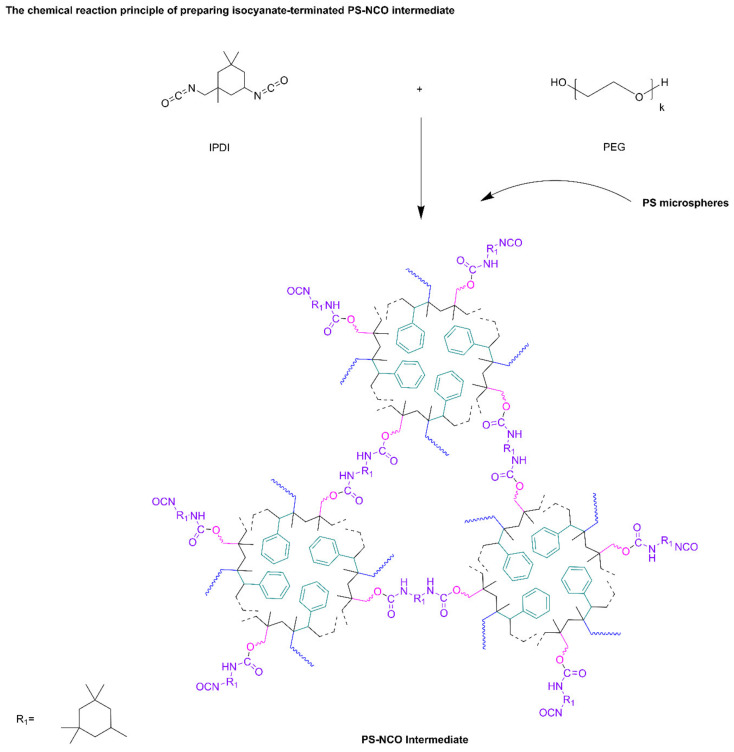
Chemical reaction principle of preparing PS-NCO.

PEG2000 and IPDI were used as the reactive monomers to conduct the initial reaction in the mixed solvent. Then the PS microspheres powder prepared in the previous step was added into the system, the –NCO in excess IPDI will react with the –OH on the surface of PS microspheres to obtain PS-NCO intermediates through in-situ polymerization, as shown in Figure 2.

3. Chemical Reaction Principle of Preparation of PS-PEG Hydrogel

**Figure 3:**
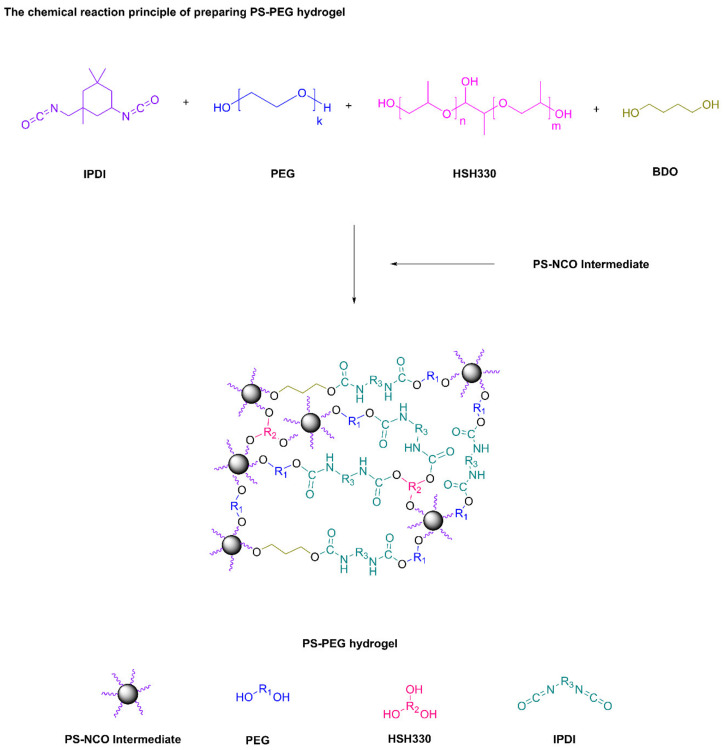
Chemical reaction principle of preparing PS-PEG hydrogel.

Using PEG2000, IPDI, and HSH330 as monomers, the isocyanate of IPDI was firstly reacted with the hydroxyl terminations of PEG2000 and HSH330 by solution polymerization method. Then PS-NCO and BDO was added into the reaction system and finally, PS-PEG hydrogel was obtained, as showed in Figure 3.

The monomer mass of PS microspheres was 7.1 wt%, 14.2 wt%, 21.3 wt%, and 28.4 wt%. The corresponding products were named as PS7-PEG, PS14-PEG, PS21-PEG, and PS28-PEG. The sample without PS microspheres is labeled as PS0-PEG.

#### 2.2.2. Technological Process

**Figure 4:**
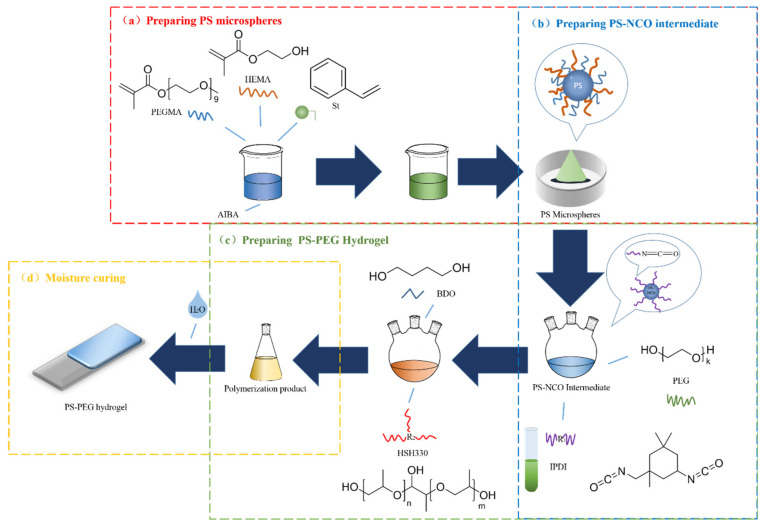
Preparation process of PS-PEG hydrogel (**a**) PS microspheres; (**b**) PS-NCO intermediate; (**c**) PS-PEG hydrogel; (**d**) Moisture curing.

The first step of the synthesis, A 500 mL four-neck flask was connected to a SZCL-2A digital intelligent control magnetic stirrer (Yuhua Instrument Co., Ltd., Gongyi, China), and 4 g HEMA, 6 g PEGMA, and 250 g deionized water were added. Meanwhile, 20 g styrene was put into 50 mL separating funnel, and control in all added into the flask about 30 min. Used DW-3 high-speed digital display electric stirrer (Yuhua Instrument Co., Ltd., Gongyi, China), and pre-emulsified at 3000 rpm for 1 h. Under the protection for nitrogen, the temperature was raised to 60 °C by using DF-101Sheat-collecting thermostatic heating magnetic stirrer (Yuhua Instrument Co., Ltd., Gongyi, China). In addition, 0.5 g AIBA was dissolved in 20 g deionized water, put into a 50 mL separatory funnel, and control about 1 h to all drip into the flask for reaction. After the content was completed, the reaction was carried out for 8 h with 280 rpm. Then, 200 mesh gauze was used to filter out the larger particles and used LC-10A-50N vacuum freeze-drying (Lichen Bangxi Instrument Technology Co., Ltd., Shanghai, China) is performed (Freeze for 12 h, dry for 24 h). Then, the powder was washed with added ethyl acetate and deionized water successively in a mass ratio of 1:1, used KQ-300E ultrasonic cleaner (Ultrasonic Instrument Co., Ltd., Kunshan, China) to wash for 40 min, and TDL-50C centrifuge (Anting Scientific Instruments Co., Ltd., Shanghai, China) was used to remove the supernatant by centrifugation for 15 min with 4000 rpm. Repeat three times to wash away the unreacted monomers. After finishing freeze-drying again (Freeze for 12 h, dry for 24 h), the powder obtained is ground and sealed, and the yield of PS microsphere powder prepared at one time was about 26 g. The specific process flow is shown in Figure 4a.

The next stage of the work, 44 g PEG was dried under reduced pressure at 120 °C for 3 h to remove water and dissolved gases. The mixed solvent (Xylene: Ethyl acetate: Cyclohexanone = 2:2:1) was filled, mixed, and stirred for 30 min, and then the temperature was raised to 70 °C. 10 g IPDI was added to the flask, reacted for 3 h, and then the temperature was lowered to 60 °C, the 10 g PS microspheres powder was slowly added, isocyanate terminated PS-NCO intermediate is obtained after 5 h, which is encapsulated and ready for use. The specific process flow is showed in Figure 4b.

Finally, 40 g PEG and 10 g HSH330 were dried under reduced pressure at 120 °C for 3 h, mixed with 80 g solvent and stirred for 30 min. 10 g IPDI was added to the flask at 70 °C and reacted for 3 h. When temperature cooled to 60 °C, 10 g PS-NCO solution was slowly added to the flask. After adding the chain extender 0.5 g BDO, the reaction ends after 5 h. The yield of PS-PEG hydrogel was about 160 g. The specific process flow is showed in Figure 4c. When it is lowered to room temperature, pour it into a polytetrafluoroethylene board for casting, and put in GDHS-2005A constant temperature and humidity box (Jinghong Experimental Equipment Co., Ltd., Shanghai, China), adjust humidity 70% RH to wet solidify to get PS-PEG hydrogel. The specific process flow is showed in Figure 4d.

#### 2.2.3. Preparation of Samples

The part of PS-PEG hydrogel solution was painted on glass slides whose dimensions are 76.2 mm × 25.4 mm × 1 mm, the rest was poured into a Teflon mold whose dimensions are 20 mm × 20 mm × 9 mm. The specimens were placed in a dust-free room temperature environment of 70% RH, cured for 7 days. The former cured was used to characterize the chemical structure and surface morphology, the latter cured to measure contact angle, swelling and compressive properties. 

### 2.3. Characterizations

#### 2.3.1. Chemical Structure

##### Fourier Transform Infrared Spectrometer (FTIR)

FTIR (EQUINOX5, Bruker, Karlsruhe, Germany) was used to analyze the chemical structure of the PS microspheres with KBr tablet method. 200 mg KBr and 2 mg PS microsphere powder were mixed and ground in an agate mortar with φ 60 mm for 1 min, and then, put into the tableting mold with φ 13 mm, pressurized to 20 MPa, and stayed for 2 min. After that, take out the mold and put the tablet into the tablet holder for infrared test. The scanning range was 4000 to 400 cm^−1^, the resolution was 2 cm^−1^, and the number of scans was 32 times.

FTIR (PERKINELMER, Waltham, MA, USA) was used to analyze the chemical structure of the PS-NCO intermediate and PS-PEG hydrogels with attenuated total reflection mode, the scanning range was 4000 to 650 cm^−1^, the resolution was 2 cm^−1^, and the number of scans was 32 times.

##### X-ray Diffraction (XRD)

XRD spectrum of the samples was performed with D/MAX-Ultima X-ray diffractometer (Rigaku Denki, Tokyo, Japan). The instrument used copper-palladium ceramics with the test range was 10–90°, the step size was 0.02°, and the time per step was set at 8° min^−1^.

#### 2.3.2. Surface Morphology

##### Scanning Electron Microscope (SEM)

The morphology of the samples was observed by Supra-55-sapphire SEM (Carl Zeiss AG, Jena, Germany). Take a square monocrystalline silicon wafer with a side length of 2 cm, and the PS microspheres emulsion was diluted and then dropped on the wafer. After being dried, liquid conductive adhesive drops are used to seal the edges around the wafers, and then the microstructure characteristics were observed by SEM. In addition, the hydrogel was prepared into a sheet of 10 mm× 10 mm× 1 mm, and adhered to the surface of the metal block with conductive tape. The JFC-1100 ion sputter (Japan Electronics Ltd., Tokyo, Japan) used direct current, adjusted the voltage and current to 5 kV and 5 mA, respectively, and sputtering on the surface of the specimen for 2 min. The observation mode is SE2, and the acceleration voltage is 1 kV. 

##### Transmission Electron Microscope (TEM)

JEOL-2100 TEM (Japan Electronics Ltd., Tokyo, Japan) was used to observe the morphology of nano microspheres. After diluting the emulsion in absolute ethanol, it was ultrasonically dispersed for 15 min, then dripped onto a copper net for air-drying, and the accelerating voltage was 200 kV.

##### Confocal Laser Scanning Microscope (CLSM)

OLS4000 CLSM (Olympus, Tokyo, Japan) was used to observe and analyze the surface and fracture of the PS-PEG hydrogels. The surface roughness (Sa) of the samples were analyzed by LEXT analysis software.

#### 2.3.3. Physical Properties

##### Differential Scanning Calorimetry (DSC)

DSC measurement was performed on NETZSCH DSC 200F3 differential scanning calorimeter (NETZSCH-Gerätebau GmbH, Selb, Germany) under a nitrogen flow of 5 °C min^−1^. The hydrogel was dried for 48 h and put into an aluminum crucible after removing the water. The scanning temperature range was set at −100–200 °C.

##### Swelling Properties

The composite hydrogel obtained after curing for 7 days were cut into cuboid specimens whose dimensions are 2.5 mm × 1 mm × 0.5 mm, and immersed in deionized water for 48 h. The samples were taken out at different time intervals, quickly dry the water on the sample surface with a filter paper, and put it into a precision balance for weighing. The swelling degree of the hydrogel SD = (WS − W0)/W0. WS and W0 are respectively the mass of the hydrogel when swelling to a certain time and primitive hydrogel.

##### Contact Angle (CA) and Surface Free Energy

CA of the hydrogels were measured by hanging drop method at room temperature using JC2000C Contact Angle Measurement Instrument (Zhongchen Digital Technology Equipment Co., Ltd., Shanghai, China), and used a syringe to drop 3 μL of the liquid (deionized water or diiodomethane) onto the surface of the sample. Meanwhile, calculating for 5 min for dynamic water contact angle (DWCA) and dynamic diiodomethane contact angle (DDCA). Owens two-liquid method [30] was used to calculate the surface free energy of PS-PEG hydrogel coating by calculating DWCA and DDCA. 

#### 2.3.4. Mechanical Properties

According to GB/T 1041–92, the hydrogel samples wet-cured for 7 days were made into cuboid mold with a length of 20 mm, a width of 20 mm and a height of 9 mm. The compression performance of hydrogels was tested by UTM 5105 computer-controlled electronic universal testing machine (Jinan Wance Electrical Equipment Co., Ltd., Jinan, China). The hydrogel sample was placed in the middle of the test bench and compressed at a speed of 1 mm/min. The elastic modulus was calculated according to the slope of the strain within 0.1 mm/mm in the stress–strain curve.

## 3. Results

### 3.1. Chemical Structure and Morphology of PS Microspheres

**Figure 5:**
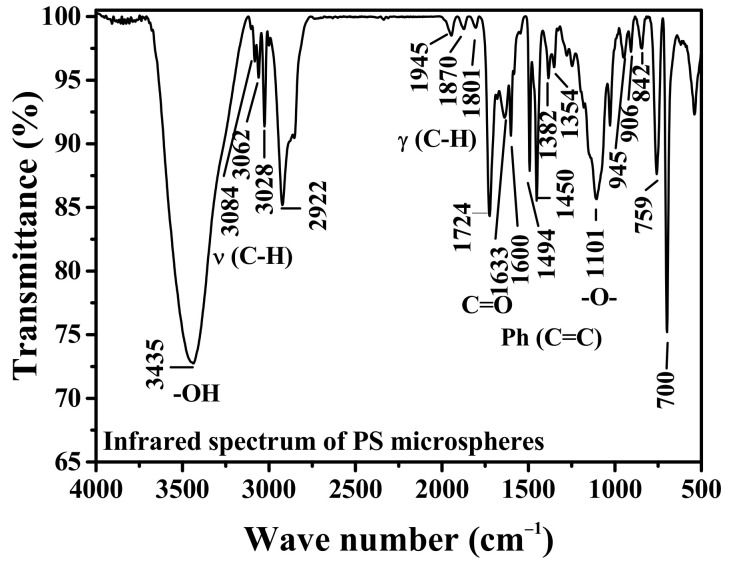
Infrared spectrum of PS microspheres.

The PS microspheres powder prepared by the freeze-drying method was tested by FTIR, as showed in Figure 5, indicating that the absorption peak at 3435 cm^−1^ was the vibration absorption peak of the hydroxyl group. The stretching vibration absorption peaks at 3000–3100 cm^−1^ were C-H bond of benzene ring. The absorption peaks at 1724 cm^−1^, 1801 cm^−1^, 1870 cm^−1^, and 1945 cm^−1^ were weak bands unique to PS, corresponding to the frequency double and group frequency absorption peaks of the out-of-plane bending vibration of the aromatic ring C-H. While the strong absorption peak at 1633 cm^−1^ was the stretching vibration absorption peak of the carbonyl group on the ester group, its original absorption peak was 1740 cm^−1^. Because of the high polarity of carbonyl group, it can form conjugate effect with the benzene ring, so that the absorption peak of carbonyl group moves to the direction of low wave number, that is, the absorption frequency decreases [31]. The absorption peaks at 1450 cm^−1^, 1494 cm^−1^, and 1600 cm^−1^ are the skeleton vibration absorption peaks of the benzene ring. The absorption peaks at 1354 cm^−1^ and 1382 cm^−1^ were the in-plane bending vibration absorption peaks of alkyl C-H, and the absorption peak of ether bond at 1101 cm^−1^. In the meanwhile, the absorption peaks at 842 cm^−1^, 906 cm^−1^, and 945 cm^−1^ were the C-H out-of-plane flexural vibration absorption peaks of the benzene ring, and the absorption peaks at 700 cm^−1^ and 759 cm^−1^ were the monosubstituted benzene ring. The above characteristic peaks indicate that PS microspheres have hydroxyl, carbonyl, and benzene ring functional groups.

**Figure 6:**
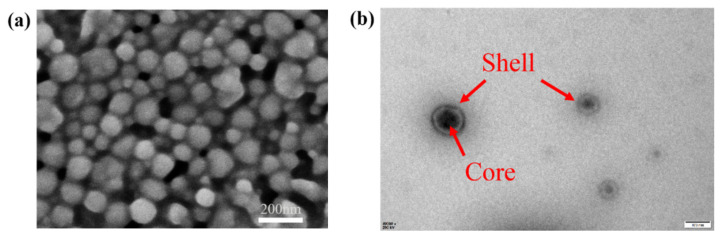
Microstructure of PS microspheres (**a**) SEM image; (**b**) TEM image.

The morphology of PS microspheres was observed by SEM and TEM. Figure 6a shows the SEM image of PS microspheres with the same scale. Clearly, PS microspheres have a smooth surface, compact arrangement, and a diameter of about 100 nm. PS microspheres are visualized by transmission electron microscopy. As shown in Figure 6b, it can be seen that that the core–shell PS microspheres have an average diameter of about 100 nm. The shell looks very uniform with a thickness of about 11 nm. The PS microspheres with styrene as core, hydroxyethyl methacrylate and polyethylene glycol methyl ether methacrylate as shell were successfully prepared by soap-free emulsion polymerization.

### 3.2. Chemical Structure and Morphology of PS-PEG Hydrogels

**Figure 7:**
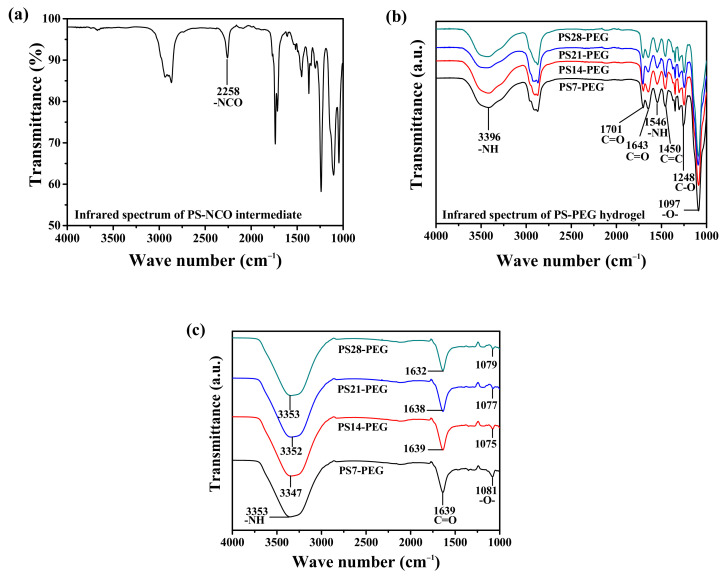
FTIR spectra of studied samples (**a**) PS-NCO intermediates; (**b**) PS-PEG hydrogel; (**c**) PS-PEG hydrogel swelling in deionized water for 48 h.

ATR-FTIR tests were carried out on the PS-NCO intermediate terminated with isocyanate through bulk polymerization and the PS-PEG hydrogel as the final product. The infrared spectra obtained were showed in Figure 7a and Figure 7b, respectively. There is a very obvious absorption peak at 2258 cm^−1^ in Figure 7a. This is the absorption peak of isocyanate (-NCO), which proves that the synthetic PS-NCO intermediate will successfully seal the isocyanate. As showed in Figure 7b, PS-PEG hydrogels with different contents were successfully synthesized. It can be observed from the infrared spectrum that the stretching vibration absorption peak of the -NH bond is located at 3396 cm^−1^, and its deformation vibration peak is at 1546 cm^−1^. In addition, at 1643 cm^−1^ and 1701 cm^−1^, the stretching vibration peak of C=O in the amide carbonyl group and the stretching vibration peak of C=O in the urea group is separated. The above characteristic peaks indicate that the final product is PEG-based hydrogel. The skeleton vibration absorption peak of the benzene ring is at 1450 cm^−1^, C-O stretching vibration peak is at 1248 cm^−1^, and the strong absorption peak at 1097 cm^−1^, is the -O- asymmetric stretching vibration peak polyether structure, which indicates that PEG-based hydrogel successfully introduces the structure containing benzene ring and polyether. Moreover, there was no obvious absorption peak between 2200 cm^−1^ and 2300 cm^−1^, indicating that the isocyanate in the polymer had been thoroughly reacted at this time, and the final synthetic substance was PS-PEG composite hydrogel.

The hydrogel sample after soaking and swelling in deionized water for 48 h, as showed in Figure 7c. The absorption peak of free -NH formed hydrogen bond with carbamate was located at 3340 cm^−1^. In contrast, the carbonyl peak C=O after soaking in different solutions was redshifted to about 1638 cm^−1^, and the ether bond region was also redshifted from 1097 cm^−1^ to 1074 cm^−1^. The main reason is that a large number of water molecules in various solutions provide the -H receptor, which increases the degree of hydrogen bonding.

The above analysis showed that the PS-PEG hydrogel was successfully synthesized. After wet curing, the free -NH and -CO- stretching vibration region were split into two peaks, namely, the free absorption peak and the absorption peak forming hydrogen bond. Meanwhile, the isocyanate was consumed, and after soaking, the -NH and -CO- on the hydrogel structure tended to form a hydrogen bond with water molecules. As a result, the number of hydrogen bonds increases, but the number of hydrogen bonds on the segment structure decreases, which weakens the degree of hydrogen bonding of the hydrogel structure chain.

**Figure 8:**
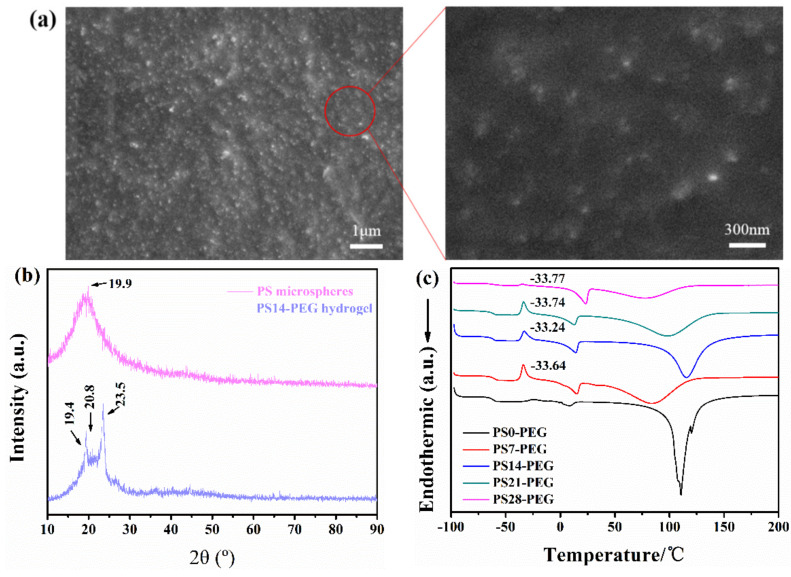
(**a**) SEM image of PS-PEG hydrogel; (**b**) XRD spectra of PS14-PEG hydrogel; (**c**) DSC curves of PS-PEG hydrogel.

SEM observation showed PS microspheres were uniformly dispersed in PS-PEG hydrogels, refers in Figure 8a. XRD spectra of PS microspheres and PS14-PEG hydrogel were shown in Figure 8b. Clearly, there was a broad peak near 2θ of 19.9°, which was a characteristic amorphous diffraction peak, and it was consistent with the literature [32]. There were two dominant characteristic peaks respectively at 19.4° and 23.5° which derived from the PEG, which were consistent with the literature [33]. However, it can be observed that the amorphous peak of PS shifted about 0.9° in the hydrogel by compared with two diffraction peaks, which due to the distortion of the crystal lattice caused by the internal residual stress of hydrogel. In this regard, DSC is further used to perform differential thermal analysis on each hydrogel sample, it can be clearly observed that the PS-PEG hydrogels added with PS microspheres all have an exothermic crystallization peak at about −33 °C in Figure 8c. In summary, combined with the infrared spectrum, the PS microspheres were successfully compounded with the PEG-based hydrogel.

**Figure 9:**
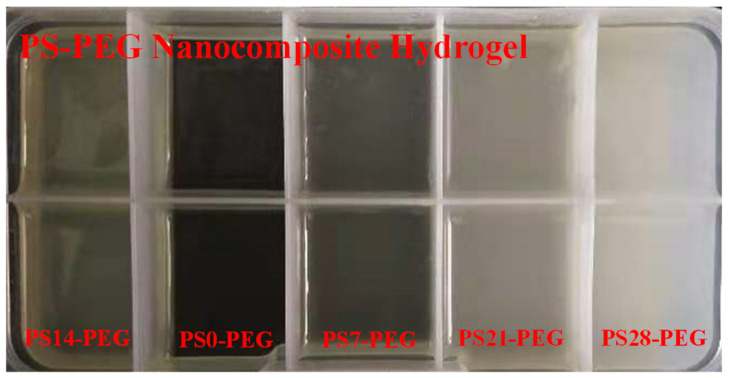
Morphology of PS-PEG hydrogel.

The morphology diagram of PS-PEG composite hydrogel with each PS microspheres addition number was clearly as showed in Figure 9. The observation results also indicated that with the increase of PS microspheres, the light transmittance of the composite hydrogel decreased gradually. While the macroscopic light transmittance of the PS0-PEG sample without PS microspheres is the strongest.

### 3.3. Swelling Properties of Hydrogels

**Figure 10:**
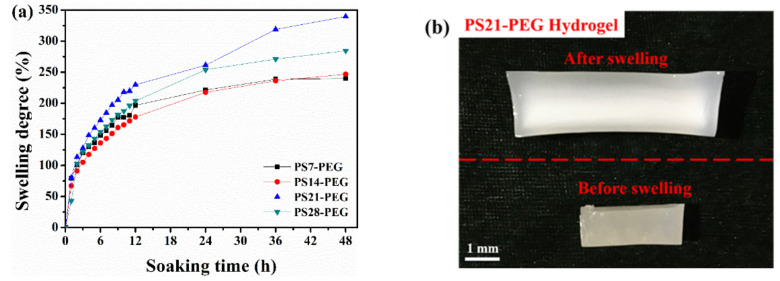
(**a**) Swelling degree with soaking time; (**b**) morphology of PS21-PEG hydrogel before and after swelling.

The swelling properties of each sample of PS-PEG hydrogel placed in deionized water for 48 h were showed in Figure 10a. In the first 3 h of the initial swelling stage, the swelling degree of each sample increased significantly in water absorption. With the increase contents of PS microspheres, PS-PEG hydrogels had rapid water absorption and swelling property in the first 6 h. Then the water absorption and swelling degree slowed down, but it still kept increasing. As the PS microspheres continued to increase, the swelling degree of the PS28-PEG sample was smaller than that of the PS21-PEG sample; that is, the swelling degree increased first and then decreased. This is because the swelling of hydrogel is determined by hydrogen bonding and hydrophobicity, and the PS microspheres contain oleophilic groups simultaneously. When the content exceeds a certain proportion, the water absorption of the hydrogen bond is less than that of its hydrophobic structure so that the swelling degree will be affected to a certain extent. At the same time, we calculated the swelling degree of each hydrogel sample after soaking for 24 h and 48 h, as listed in Table 1. It demonstrates that the PS21-PEG sample had the highest swelling degree, not the PS28-PEG, which is because the dense three-dimensional cross-linking structure is formed after the third step of polymerization. However, the limited space of the system will further hinder the absorption of water by the hydrogel.

The volume of PS21-PEG hydrogel after swelling for 48 h increases to about 3.4 times that of original dry gel sample, as shown in Figure 10b. It can be observed from the figure that the volume of the hydrogel has undergone macroscopic expansion after swelling. It always keeps a certain swelling degree in deionized water. This is because the -OH of the PS shell layer with a core–shell structure can be cross-linked with -NCO, making the PS microspheres a ‘central connection point’, which promotes the overall hydrogel the three-dimensional structure was significantly improved.

### 3.4. Contact Angle and Surface Energy

Since static contact angle is measured under equilibrium condition, it can only reflect the wettability at equilibrium, and cannot reveal the change information of surface structure. It is unable to study the relationship between material surface structure and wettability, as well as the precise control of the surface structure. However, dynamic contact angle just makes up this shortcoming. It can provide information on surface roughness, uniformity of chemical properties, reconstruction of hydrophilic/hydrophobic chain segments. The dynamic water contact angle (DWCA) and dynamic diiodomethane contact angle (DDCA) of PS-PEG are calculated for 5 min, respectively. The surface free energy is calculated as listed in Table 2.

**Figure 11:**
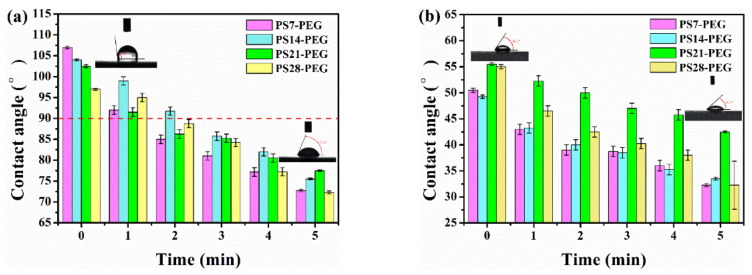
PS-PEG hydrogel dynamic contact angle (**a**) DWCA; (**b**) DDCA.

PS-PEG hydrogel was tested in deionized water for DWCA, as showed in Figure 11a. The initial DWCA was PS7-PEG > PS14-PEG > PS21-PEG > PS28-PEG. The final state DWCA was PS21-PEG > PS14-PEG > PS7-PEG > PS28-PEG. As the number of PS microspheres increased, the initial DWCA became lower and changed from hydrophobic to hydrophilic within 5 min (from 104° to 75.5° in the case of PS14-PEG). This was because the PS surface of the PS-PEG hydrogel compound with PS microspheres powder was rich in a large -OH, which was a strong hydrophilic group. In the subsequent synthesis process, after the reaction with isocyanate and wet solidification of the film, the chain segment formed by it is more flexible. Therefore, for the test area of water droplets, the more flexible hydrophilic chain segment is more likely to migrate and enrich on the surface of the coating, realizing the conversion process from hydrophobic to hydrophilic, and then forming a layer of hydration film. Therefore, it appears to be strongly hydrophilic after the appearance of hydrophobicity. It also can be observed from the swelling test.

The results after the DDCA test are plotted as showed in Figure 11b. It is not difficult to observe that the initial DDCA is PS21-PEG > PS28-PEG > PS7-PEG > PS14-PEG. The final DDCA is PS21-PEG > PS14-PEG > PS7-PEG > PS28-PEG. All samples are lipophilic (take PS14-PEG as an example, from 49.25° to 33.5°). In addition, DWCA and DDCA showed the same change trend. PS-PEG hydrogels has both hydrophilic and lipophilic, when the surface contacts with polar or non-polar media, internal structure of the hydrogel will be restructured within a certain period of time, and the corresponding functional groups will eventually accumulate on the surface through the turnover of the flexible chain.

The surface energy of the coating was calculated according to the given formula by measuring the water contact angle and diiodomethane contact angle. The atoms (or ions) on the surface are in a state of imbalance between internal and external forces. The more unbalanced the force is, the more unstable it is and the higher its surface energy is. Therefore, as evidenced by the surface energy of hydrogel can be reduced by adding PS microspheres properly, and the surface energy decreased with the increase of PS microspheres. The surface of PS28-PEG sample is minimum, only 31.45 mJ/m^2^.

### 3.5. Surface Morphology and Roughness

**Figure 12:**
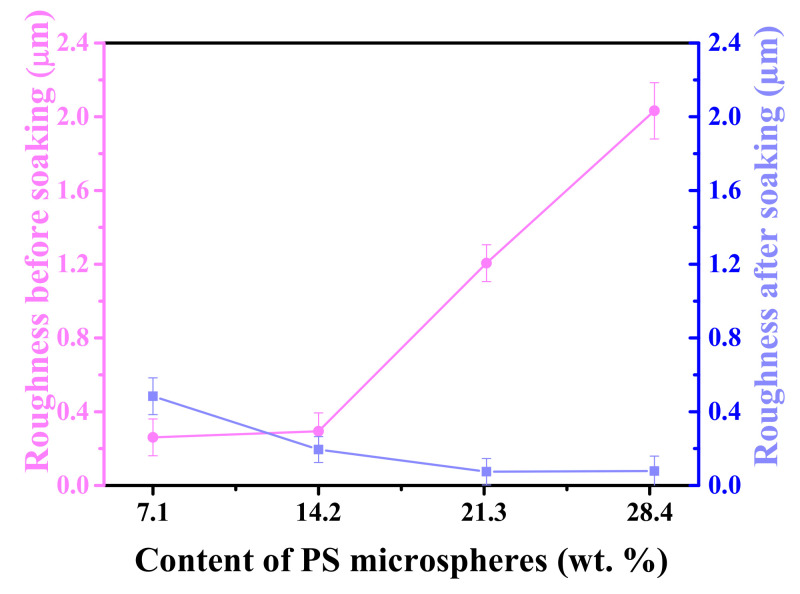
Roughness of PS-PEG hydrogels before and after swelling for 48 h.

The relationship between the surface roughness of the coating before and after soaking and the content of PS microspheres was showed in Figure 12. The surface roughness of the hydrogel before immersion increased with increasing the content. This is because the nano-PS microspheres have high surface activity. Therefore, too many nanospheres will increase the agglomeration effect. The agglomeration effect is enhanced, small agglomerations are easily formed in the partially incompletely dispersed areas, which increases the surface roughness.

When the sample swelled for 48 h, the surface roughness of the sample decreased with the increase of PS microspheres. This is because PS microspheres are used as the crosslinking points in the hydrogel, making the crosslinking density increase, it caused three-dimensional space to become more compact when the hydrogel absorbs water, it can form a hydration layer on the surface, and the roughness of the surface is reduced. Therefore, the surface of the hydrogel after swelling at the same time is more even and smooth. As for PS14-PEG hydrogel with PS microspheres added number of 14.2 wt%, the roughness of PS14-PEG hydrogel was not only low before soaking, but also decreased significantly after soaking for 48 h, which was better than that of other samples.

### 3.6. Compression Performance 

The rheological test cannot reflect the macroscopic fracture property of the material under large deformation, so it is necessary to test the compression property of the hydrogel.

**Figure 13:**
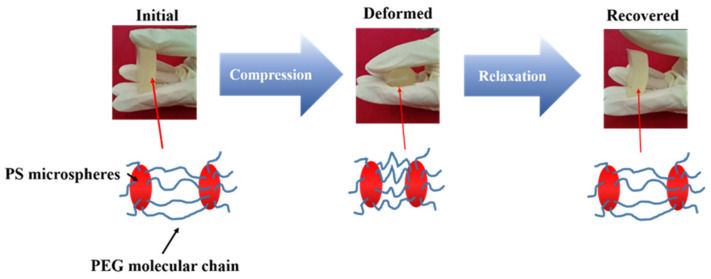
Schematic diagram of compression deformation and recovery of PS-PEG hydrogel.

The results showed that a simple compression test of PS-PEG hydrogel, as showed in Figure 13. It was observed that the hydrogel can recover to its original shape after the removal of external force without being damaged, with certain elasticity.

**Figure 14:**
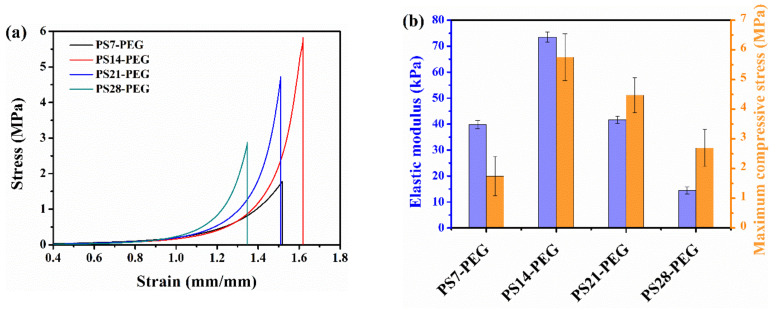
Mechanical properties of PS-PEG hydrogels (**a**) compressive stress–strain curves; (**b**) elastic modulus and maximum compressive stress.

The compressive stress-strain curves, compressive elastic modulus and maximum compressive stress of all samples were showed in Figure 14a,b. Obviously, the maximum compression stress of PS14-PEG hydrogel reached 5.83 MPa, which was nearly 3.3 times higher than that of PS7-PEG hydrogel 1.78 MPa. However, the maximum compression stress gradually decreased with increasing PS microspheres, and the maximum compression stress of PS28-PEG hydrogel was 2.89 MPa. The maximum compression stress of PS28-PEG is 2 times lower than that of PS14-PEG. The maximum compressive stress of PS14-PEG hydrogel is much higher than that reported by Jiang [34]. He used hyperbranched polyethyleneimine (HPEI) as the main material and prepared the quaternary ammonium nanoparticles, these nanoparticles were added to HPEI to prepare hydrogels. The maximum compressive stress of his hydrogels reached nearly 2.5 MPa.

Similarly, the relative compression ratio increased first and then decreased with the increase of PS microspheres. The relative compression ratio of PS14-PEG hydrogel reached 161.96%, while PS21-PEG and PS28-PEG were damaged at 151.04% and 134.71%, respectively. This indicates that the proper content of PS microspheres can effectively improve the compression performance of the composite hydrogels. However, the excessive PS microspheres will increase the density of hydrogen bonds and crosslinking points between the segments in the hydrogel. The increasing density of overall three-dimensional network structure makes chain segments difficult to move.

The compressive elastic modulus was fitted by the stress-strain curve. With increasing the content of PS microspheres from 7.1 wt% to 14.2 wt%, the maximum compression modulus increased from 39.6 kPa to 73.7 kPa, the latter was 1.86 times higher than the former. Afterwards, the compressive modulus of the hydrogel decreased gradually, and the PS28-PEG hydrogel was only 14.8 kPa. Compared with PS14-PEG, the reduction was nearly five-fold. The results showed that too many PS microspheres can easily lead to their agglomeration, this leads to uneven distribution of internal structures. Too many rigid PS microspheres reduced the elastic modulus and compressive strength of hydrogels.

The dispersion of low-concentration nanoparticles was relatively uniform. The molecular chain between two nanoparticles was longer, so the degree of molecular chain curl was higher. A large number of energy were consumed in the compression process. Therefore, the PS-PEG hydrogels exhibit some elasticity at low concentrations. With the increase of PS microspheres content, the cross-linking density of hydrogel increased, it resulted in a shorter chain between the two nanoparticles, so the elasticity goes down. At this time, when the hydrogel is extruded by compression, the space size of the microspheres were irreversibly destroyed to a certain extent.

## 4. Discussion

**Figure 15:**
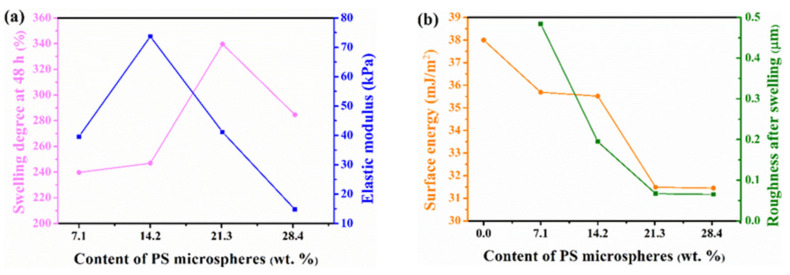
Changes in various properties with the content of PS microspheres (**a**) swelling degree and mechanical properties; (**b**) surface energy and roughness.

The above results demonstrate that the content of PS microspheres not only affects the surface properties of composite hydrogels, but also affects the physical properties and mechanical properties of composite hydrogels. With the increase of the content of PS microspheres, the mechanical properties of the hydrogel gradually increase, as showed in Figure 15a. when the content of PS microspheres reaches 14.2 wt%, the mechanical properties of the hydrogel are the maximum, while the corresponding the swelling degree was lower at 48 h, indicating that the internal cross-linking structure of the hydrogel is the tightest; while continuing to increase the number of PS microspheres added, the mechanical properties show a downward trend. In addition, the surface roughness and surface energy of the hydrogel decreased with the increase of PS microspheres, as showed in Figure 15b.

**Figure 16:**
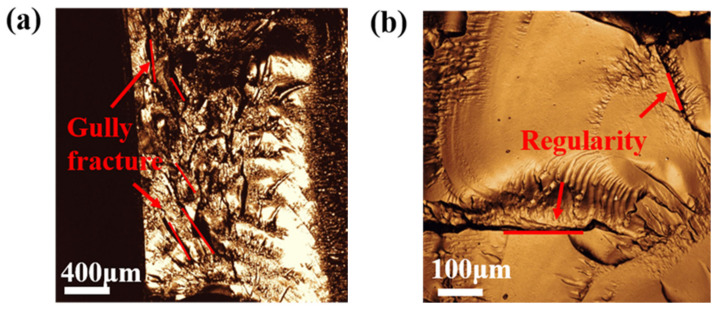

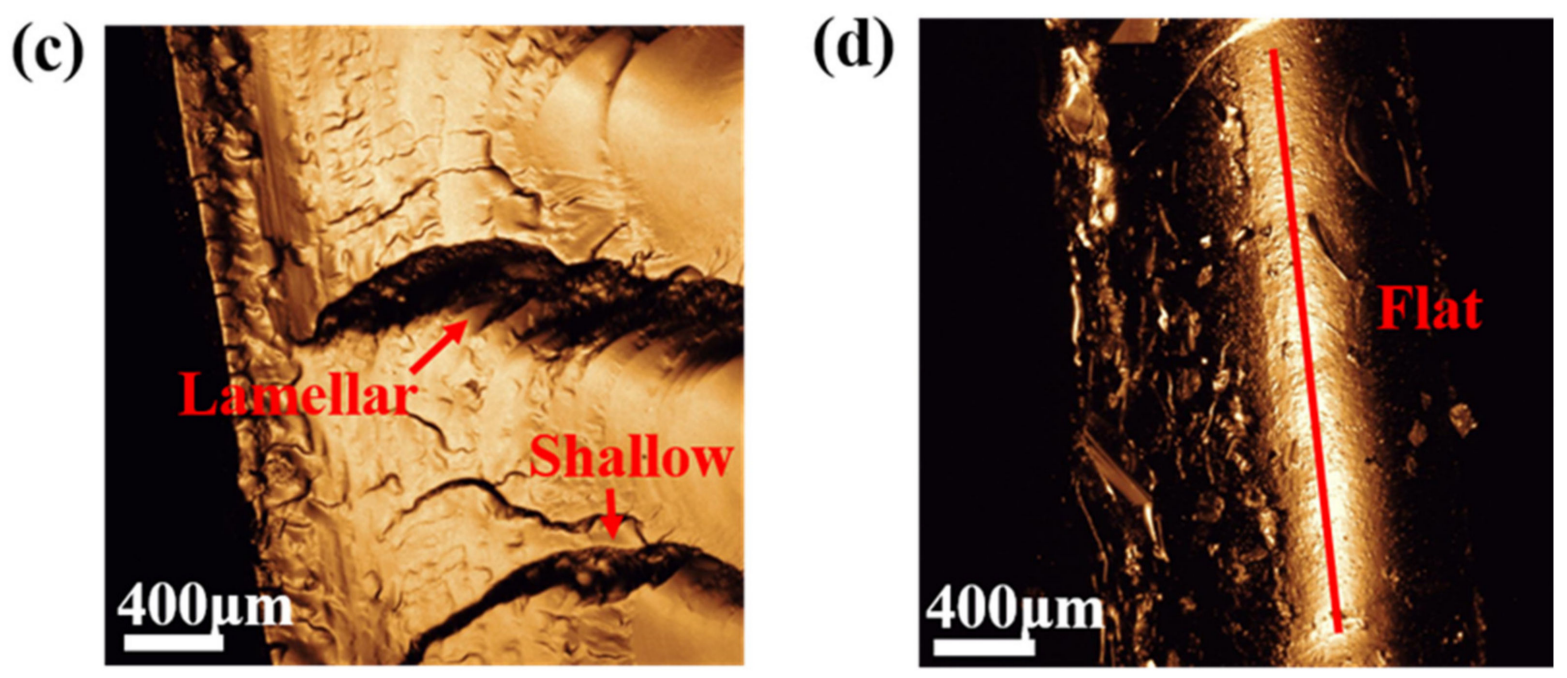
Fracture morphology of PS-PEG hydrogels. (**a**) PS7-PEG. (**b**) PS14-PEG. (**c**) PS21-PEG. (**d**) PS28-PEG.

The deep-frozen fracture morphology of hydrogel samples was shown in the Figure 16. The observation indicates that the content of PS microspheres obviously affect the fracture morphology of the hydrogel. The fracture of PS7-PEG hydrogel has a certain arrangement of gully shape, as showed in Figure 16a. This is because a few PS microspheres can make the three-dimensional network crosslinking structure of the hydrogel more compact. However, the number of connection points is limited and the crosslinking structure is not perfect, so the segment with weak bonding strength breaks when subjected to external stress. When the number of microspheres continued to increase, the fracture groove of PS14-PEG hydrogel was significantly less than that of PS7-PEG hydrogel, as showed in Figure 16b, and has a certain regularity, is not along the only direction of fracture. This is because the hydrogel will deform under the action of natural forces, in the place where the spatial structure of hydrogel is cross-linked closely, the bond strength is high and it is not easy to be broken, while in the place where the relative bond strength is low, it is easy to reach the limit and break. The fracture occurs in the position where the relative strength of the hydrogel bonding is poor, so the whole fracture process will be affected by the distribution characteristics of this region. Therefore, the fracture position is different at this time, and it has a certain spatial structure regularity.

**Figure 17:**
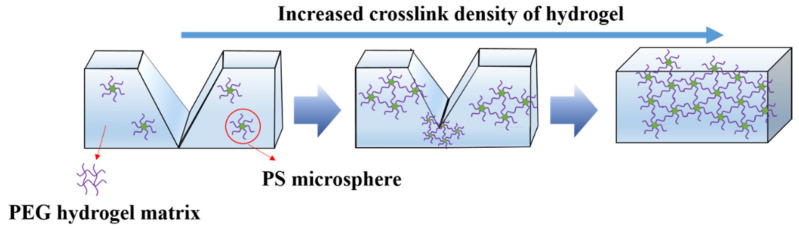
Influence of PS microspheres content on fracture strength.

Continue to increase PS microspheres, as showed in Figure 16c, the PS21-PEG hydrogel cross section is similar to lamellar fault, and the depth of the gully produced by the fracture cracks is relatively shallow. The reason is that the internal crosslinking structure continues to increase and the binding strength increases with the further increase of the content number of nano-spheres. Therefore, in the whole process of fracture, the strength of longitudinal fracture cannot extend well to the interior, it only keeps the regular fracture in the transverse direction. The corresponding PS28-PEG hydrogel had the most PS microspheres, as showed in Figure 16d. The results demonstrates that its fracture morphology was relatively flat, and there was no obvious fracture layer and similar gully shape. This was attributed to the fact that the sample contains the most PS microspheres, and the full cross-linking reaction inside the hydrogel results in the highest binding strength, thus forming a three-dimensional structure with high strength, which delays the further fault damage of the internal structure caused by the external force. Consequently, the influence of PS microspheres content on fracture strength can be generalized schematically as showed in Figure 17.

## 5. Conclusions

(1) PS microspheres with a core–shell structure were successfully prepared by soap-free emulsion polymerization. The diameter of the microspheres was about 100 nm and the shell thickness was about 11 nm. Moreover, PS microspheres reinforced PEG-based hydrogels were successfully prepared by bulk polymerization and solution polymerization.
(2) The swelling degree of PS-PEG hydrogel changes with the content of PS microsphere. When its content was 14.2 wt%, its swelling degree was 246.9% at 48 h. PS-PEG hydrogels have good swelling performance.
(3) The static water contact angle and surface free energy of the hydrogels decreased with increasing PS microspheres content, but diiodomethane contact angle creased. The surface energy of PS28-PEG hydrogel is only 31.45 mJ/m^2^. Moreover, the surface of PS-PEG hydrogel can changed from hydrophobic to hydrophilic during the wetting process.
(4) The content of PS microspheres can affect the surface roughness of the hydrogel. The surface roughness of the dry gel increases with the increase content of microspheres. However, the surface roughness of the soaked PS-PEG hydrogel decreased with the content of PS microspheres.
(5) Appropriate content of PS microspheres can improve the mechanical properties of PS-PEG hydrogels. When the content of PS microspheres is 14.2 wt%, the maximum compressive stress reaches 5.83 MPa, the relative compression ratio reaches 161.96%, and the compressive elastic modulus is 73.7 kPa.

## Figures and Tables

**Table 1:** Swelling degree of studied samples.

Sample	PS7-PEG	PS14-PEG	PS21-PEG	PS28-PEG
Swelling degree at 24 h (%)	221.51	217.70	261.34	253.85
Swelling degree at 48 h (%)	239.78	246.90	339.64	284.62

**Table 2:** WCA and DCA and surface free energy of PS- hydrogel samples.

Sample	WCA (°)	DCA (°)	Surface Free Energy (mJ/m^2^)
PS7-PEG	107 ± 0.33	50.5 ± 0.41	35.52 ± 0.19
PS14-PEG	104 ± 0.24	49.25 ± 0.33	35.69 ± 0.16
PS21-PEG	102.5 ± 0.36	55.5 ± 0.28	31.49 ± 0.13
PS28-PEG	97 ± 0.22	55 ± 0.42	31.45 ± 0.24

## Data Availability

The data in this study are contained within the article.
